# Strengthening Critical Health Literacy for Health Information Appraisal: An Approach from Argumentation Theory

**DOI:** 10.3390/ijerph18136764

**Published:** 2021-06-23

**Authors:** Sara Rubinelli, Alexander Ort, Claudia Zanini, Maddalena Fiordelli, Nicola Diviani

**Affiliations:** 1 Department of Health Sciences and Medicine, University of Lucerne, 6002 Luzern, Switzerland; alexander.ort@unilu.ch (A.O.); claudia.zanini@paraplegie.ch (C.Z.); nicola.diviani@paraplegie.ch (N.D.); 2 Swiss Paraplegic Research, 6207 Nottwil, Switzerland; maddalena.fiordelli@usi.ch; 3 Institute of Public Health, Università della Svizzera Italiana, 6900 Lugano, Switzerland

**Keywords:** health information, disinformation, misinformation, health literacy, critical health literacy, health communication, argumentation theory, information appraisal, critical thinking

## Abstract

The overload of health information has been a major challenge during the COVID-19 pandemic. Public health authorities play a primary role in managing this information. However, individuals have to apply critical health literacy to evaluate it. The objective of this paper is to identify targets for strengthening critical health literacy by focusing on the field of argumentation theory. This paper is based on the textual analysis of instances of health information through the lens of argumentation theory. The results show that critical health literacy benefits from: (1) understanding the concept of argument and the supporting reasons, (2) identifying the main argument schemes, and (3) the knowledge and use of the main critical questions to check the soundness of arguments. This study operationalizes the main aspects of critical health literacy. It calls for specific educational and training initiatives in the field. Moreover, it argues in favor of broadening the current educational curricula to empower individuals to engage in informed and quality decision making. Strengthening individuals’ critical health literacy involves interventions to empower in argument evaluation. For this purpose, argumentation theory has analytical and normative frameworks that can be adapted within a lay-audience education concept.

## 1. Introduction

The recent COVID-19 pandemic and the related mushrooming of fake news, conspiracy theories, and more general disinformation has put the so-called “infodemic” in the global spotlight. The WHO refers to infodemic as the overload of information, including mis/disinformation [1]. Due to the increased discourse around those issues, one could get the impression that the start of the COVID-19 health crisis also caused the infodemic. However, the phenomenon is not new and dates back at least two decades to the advent of the Internet in the late nineties [2]. The introduction of this new technology also led to the extensive availability of health-related information, which goes hand in hand with low-threshold accessibility to everyone with an internet connection. Regardless of the vast benefits, online health information—because of its uncontrollable nature—also has the potential to have detrimental effects on citizens and society as a whole. For example, citizens, especially those with a limited ability to distinguish between good and bad information, could take crucial health-related decisions based on wrong or incomplete information, consequently putting themselves and others at risk of suboptimal health outcomes [3]. A concrete example in the context of the COVID-19 pandemic is the abundance of fake news surrounding face masks; different sources have claimed that they do not protect from the virus or even that they are unhealthy. Such disinformation can lead to detrimental outcomes, as people following such claims are less likely or would entirely refrain from wearing a face mask, with possible dire consequences [4,5,6].

Public health authorities can play a central role in this context [7]. One possible solution to counter the respective risks would be to design systems that allow monitoring and contain the spread of mis/disinformation. Additionally, many endeavors in this direction are now ongoing at the national and international level, such as the World Health Organization’s “Call for Action: Managing the infodemic” [8]. However, due to the nature and magnitude of the phenomenon, it is unlikely to identify and control all the information available, especially online. Therefore, it is crucial to equip individuals with the necessary set of skills to understand and evaluate health information or, in other words, to distinguish between information and disinformation. This set of skills is referred to as *critical health literacy* [9,10,11]. Although the potential of critical health literacy is now widely recognized by researchers and policymakers, its operationalization is still in its infancy, thus hindering the development of targeted interventions [12].

This paper aims to identify targets for strengthening an individual’s critical literacy about the appraisal of health information. This objective is achieved by theorizing and illustrating some main principles for evaluating information rooted in the field of *argumentation theory* and by contextualizing them from a health-educational perspective.

## 2. Analytical Approach

Argumentation theory is conceptually and empirically well developed [13]. Specifically, “argumentation theory”, in its very essence, is the body of knowledge that deals with the study of arguments, that is, of claims supported by reasons [14,15,16,17]. For example, in the sentence, “these flowers are beautiful because they have beautiful colors”, “these flowers are beautiful” is the claim, and “they have beautiful colors” is the reason that supports the claim. In generalized terms, argumentation theory studies *how* to support claims by reasons and *whether this support is sound or unsound*. Still, little attention has been paid to harness its main achievements: the potential to guide and educate people in the process of appraising health information. This is unfortunate, as taking the essence of this knowledge into account, argumentation theory can provide valuable insights on how to guide people in the process of evaluation, i.e., whether a particular piece of health information contains arguments and whether these arguments are qualitatively high or poor [18,19]. Consequently, information containing poor-quality arguments should be questioned because, as we shall see, it could be false, irrelevant, or manipulative [20]. 

To fill this gap, this paper applies the approach of textual analysis to the main theoretical frameworks from argumentation theory. It analyzes examples of arguments—inspired by actual instances of dis/misinformation that actually circulated during the global COVID-19 health crisis—with the goal to pointing towards ways to identify low-quality health information. In doing so, the following sections will explain the value of the following argumentation theory concepts: the structure and purpose of arguments, argument schemes, fallacies and critical questions. Implications of the analysis will then been discussed in the context of critical health literacy.

## 3. Insights from Argumentation Theory

### 3.1. The Structure and Purpose of an Argument

As anticipated above, argumentation is a process of communication where the speaker supports their point of view (a claim) by means of reasons. Although among English speakers, the term “argument” often connotes “quarrel”, its technical meaning, at the basis of argumentation theory, indicates the process and act of discussing with reasoning for or against a claim. For instance, the claim “people should wear masks” might be supported by the reasons “because it is a key measure for suppressing the transmission of COVID-19 (reason 1) and for saving lives (reason 2)”.

**Key insight 1: People should be made aware of the concept of argument and that the acceptance of a claim derives from the approval of the supporting reasons that, as such, need to be verified and evaluated.**

People make and present claims and arguments because they want to convince an audience to think or act in a certain way. A health promotion institute can present arguments to convince people to wash their hands frequently during the day. However, due to vested interests, one might try to convince an audience that COVID-19 is like the flu and provide their reasons for this (for instance, that “he/she is an expert and knows about that”).

Although, as we shall see below, reasons can be false, wrong, or misleading, the fact that a speaker provides them to support their claim is an important way to verify the quality of the claim itself [21]. Indeed, if someone says “Don’t wear a mask” without saying why the claim is weak in the sense that the rationale behind it is unclear. If the speaker says, “Don’t wear a mask (claim) because COVID-19 is no worse than the flu (reason 1) and masks are even hazardous as they impair breathing (reason 2)”, reasons 1 and 2 offer grounds for verifying the claims and for deciding whether one should believe in it. Then, of course, the reasons provided are not sound. However, to carry out this evaluation, the concept of arguments has to be clear in people’s minds, as well as the supporting reasons and the need to check their quality. In the discussion section of this paper, we shall highlight how this presupposes the daily work of health institutions in presenting and discussing evidence so that people have quality sources to evaluate the reasons behind claims.

A primary skill in the context of critical health literacy and information appraisal is to check whether or not the claims put forward present reasons and, subsequently, whether or not these are valid and supported. Indeed, in the current world of influencers and opinion leaders, it is not unusual that people believe in claims that are unsupported simply because they trust the speaker. The Elaboration Likelihood Model [22] shows that, indeed, people might appraise information and arguments within a peripheral perspective, focused on aspects that do not point to the goodness of a claim. To agree that “masks kill” simply because the person who says this receives trust within a particular community is a pitfall in critical thinking skills. Additionally, evaluating the soundness of argumentation based on trust in the speaker and without paying proper attention to the actual content of what he/she says is problematic: It is an evaluation based on a peripheral cue and, thus, potentially misleading. Even if the claim comes from a famous health professional, it is still unsupported due to a lack of substantiating scientific data. Moreover, the experience of a person, even if they are perceived as an expert, is not sufficient to substantiate a general claim. Especially if, such as in this case, there is scientific evidence supporting the use of masks during the pandemic.

### 3.2. Argument Schemes

The literature on argumentation theory has identified *argument schemes* as central components of an argument structure, which is crucial for evaluating argument strength. Specifically, an argument scheme is a template that indicates a specific connection between the claim and the supporting reason/s. Each argument scheme has a name [23].

**Key insight 2: People should be able to recognize the most essential argument schemes to evaluate the arguments themselves.**

According to pragma-dialectics, among the most potent approaches to the study of argumentation, a threefold typology of arguments exists [24], which we exemplify below through suboptimal information about COVID-19. Starting from an analysis of the argument schemes, we will then show how it is possible to identify why this information is of low quality.

The first argument scheme is named *symptomatic argumentation*, which poses that a claim can be supported by citing in its reasons a particular sign, symptom, or distinguishing mark of what is claimed. For example, in the following argument:

“This person is right about COVID-19 because he is a doctor.”

The fact that this person is a doctor is presented as a sign of the quality of what he says about COVID-19.

The general argument scheme for this type of argument is:
      Y is true of X,
       *because*: Z is true of X
       *and*: Z is symptomatic of Y.

In the context of the example:
      This person (X) is right about COVID-19 (Y).
       *because:* This person (X) is a doctor (Z).
       *and:* Being a doctor is a sign of knowing about health conditions, resulting in true health-related statements.

**Figure 1:**
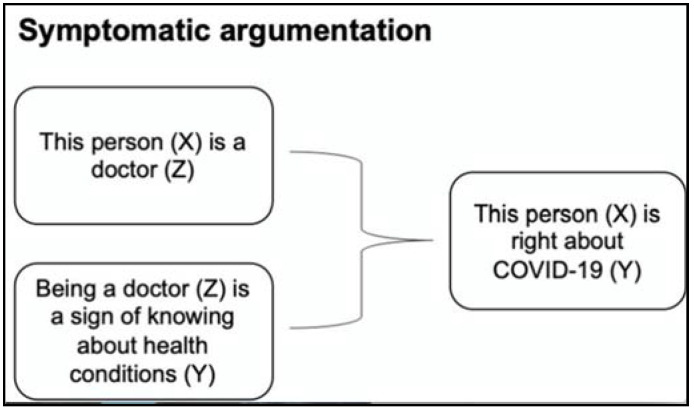
Example of symptomatic argumentation.

Figure 1 below shows the example in a visual chart:

The second type of argument schemes is based on a *comparison relation*, meaning that a claim is supported by showing that something similar occurs elsewhere. Therefore, people should also accept it for resemblance. For example, in the following argument:

“The COVID-19 vaccine is dangerous because past vaccines have also been found to be dangerous.”

Here, the comparison with other vaccines is a reason for supporting the danger of the COVID-19 vaccine.

The general argument scheme for this type of argument is:
      Y is appropriate for Y,
       *because*: Y is appropriate for Z
       *and*: Z is comparable to X.

In the context of the example:
      The COVID-19 vaccine (X) is dangerous (Y)
       *because:* past vaccines (Z) are dangerous (Y)
       *and:* past vaccines (Z) are comparable to the COVID-19 vaccine (X).

**Figure 2:**
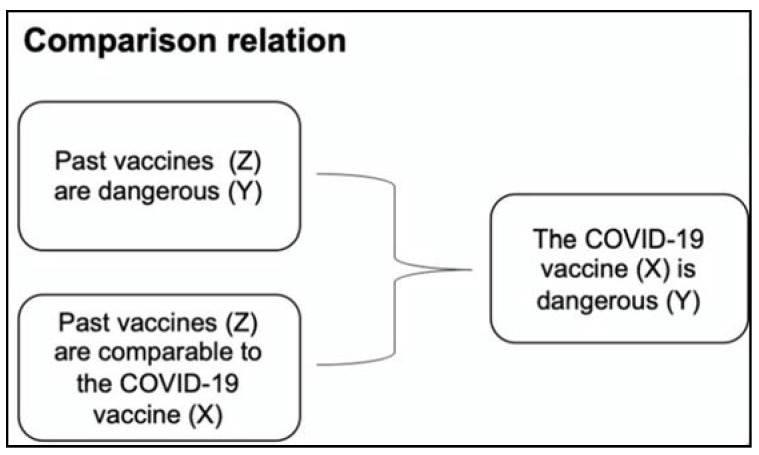
Example of argument based on a comparison relation.

Figure 2 below shows the example in a visual chart.

The third type of argument scheme is based on a *causal relation*, where the claim is supported by making a causal connection between itself and its grounding reason. For example, in the following argument:

“Some people contracted COVID-19 because they used 5G technologies.”

The general argument scheme is:
      Y is true of X,
       *because*: Z is true of X
       *and*: Z leads to Y.

In the context of the example:
      Using 5G technologies (Z) leads to contracting COVID-19 (Y).
       *because*: Some people (X) contracted COVID-19 (Y)
       *and*: Some people (X) used 5G technologies (Z)

**Figure 3:**
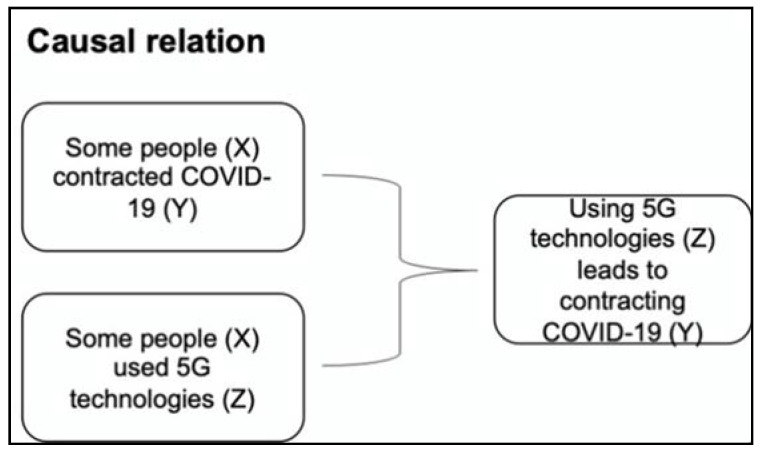
Example of argument based on a causal relation.

Figure 3 below shows the example in a visual chart.

Highlighting the formal structure of arguments seems to be technical. Yet, these three main types of argument schemes can be used to analyze and evaluate all types of arguments, where claims support reasons. Strengthening people’s skills in understanding and using these argument schemes is, thus, important as a base to avoid taking claims for granted or relying on peripheral cues that can be misleading.

### 3.3. Fallacies and Critical Questions

Identifying the structure of arguments in terms of their claims and supporting reasons is the essential step to evaluating the quality of the arguments themselves and defining whether they are of good or poor quality. When, indeed, the reasons put forward to support a claim are false, irrelevant, or, in general, not appropriate, the argument itself lacks soundness. The literature refers to these as “fallacies” that are invalid or faulty reasoning [25]. It is not always easy to recognize fallacies. Yet, the primary step is to instruct people on how to detach this type of disinformation.

**Key insight 3: People should learn to identify the structure of an argument and then verify if it is fallacious by asking critical questions.**

More specifically, people can identify the weaknesses of arguments by using “critical questions”, that is, questions to test the soundness of arguments [26]. Referring to the above-mentioned reasoning:

“This person is right about COVID-19 because they are a doctor.”

Taking into account the above-introduced knowledge about argument schemes, the main critical question to ask here is: “is Z indeed symptomatic of Y?”, that is, is being a doctor indeed a sign of knowing about all health conditions and, thus, always making factual health-related statements? The unreflected answer might be, “yes, of course”. However, the framework gets shaky by taking a closer look at the claim and the specific situation. First of all, doctors are experts about *specific* health conditions, not about *all* health conditions. Moreover, they might be right in diagnosing and treating cases that they have experience in, but not where they have no clinical experience and have not conducted research, which would hold for most doctors and COVID-19. Moreover, experience is often contextualized in a specific setting. However, for generalizations regarding, for instance, the country-specific COVID-19 clinical situation, they have to rely on national data and conduct sophisticated research that goes beyond their personal opinions. Certain aspects about COVID-19 are not within the domain of general practitioners but rather lie within the field of epidemiologists, virologists, public health experts, and health economics experts. Being a medical doctor does not directly qualify to hold expertise in other areas, even if significant overlaps exist between different health-related fields. Thus, the “authority-oriented” perspective for the argument is weak when the speaker might not have any absolute authority, knowledge, or experience in what they claim.

Concerning the second example:

“The COVID-19 vaccine is dangerous because past vaccines have also been found to be dangerous.”

Here, there are different critical questions to ask. The first question relates to the grounds for claiming that “vaccines in the past were shown to be dangerous”. Is this true? What does the evidence say and prove? Further, another important question is, “what does it mean to say that a vaccine is dangerous”? Related to these questions, the list of other questions includes: does the vaccine have some side effects like any other drug? Does it have more side effects? Have these effects been proven through rigorous scientific studies? Moreover, provided that some vaccines produced side effects in the past: Is the COVID-19 vaccine comparable to them? In other words, going back to the previous argument schemes: is Z (the COVID-19 vaccine) really equivalent to X (the other vaccines)? Is X (the other vaccines) really Y (dangerous)?

Overall, arguments from analogies always work by claiming comparisons. It is, thus, essential to verify the nature of the comparison itself. In particular, whether there is a relation/correspondence between two things and, if so, in which way.

Lastly:

“Some people contracted COVID-19 because they were exposed to 5G radiowaves.”

The main critical question is: “does Z indeed lead to Y?”, that is: does exposure to 5G lead to COVID-19? There is no scientific evidence that supports any link between the two as such. Thus, the argument is unsound, as the proposed cause of something is unproven. This is a typical pitfall of arguments implying that “something has the inevitable result”, that “something causes something”, and that “something always occurs when …”. The foundation of these general claims has to be carefully reviewed, as making faulty causal links between events indicates flawed critical thinking, which can lead to dangerous health decision-making.

## 4. Discussion

This paper contributes to the field of critical health literacy. It proposes specific topics from argumentation theory that could inform the operationalization of critical health literacy and form a basis for interventions aiming at strengthening individuals’ skills in this context. This study argues that a promising way to empower people in evaluating health information is to enhance their skills in the recognition, analysis, and evaluation of arguments. Specifically, it supports the need for people:

(1) to recognize when health information is argumentative, that is, when the speaker presents a claim that they want the audience to accept, believe, or act upon;
(2) to identify whether reasons support claims, and if so, which argument scheme they implement (that is, schemes based on symptomatic relations, analogy, or causality);
(3) to ask the main critical questions to look for evidence behind the proposed reasons that can support or deny the claims’ validity (and acceptance).

These insights stemming from argumentation theory can contribute to advancing research on critical health literacy and align with existing findings in these contexts. An individual’s ability to recognize arguments and distortions in information has indeed been identified as one of the central components of critical health literacy in a recent review aiming to operationalize the concept [10]. Always keeping in mind that critical appraisal of information is complex and also involves a variety of competencies, skills, and abilities in other contexts (e.g., the ability to recognize biases in one’s thinking), this analysis based on argumentation theory provides us with concrete examples of what these distortions could look like and how to identify them. This makes our contribution a precious addition for the conceptualization and operationalization of critical health literacy. Despite its mainstream relevance in today’s information landscape, research in this domain is still in its infancy [12].

Moreover, some main considerations are needed when thinking about the applicability of our proposed approach. First, we are very well aware that argumentation theory is a technical discipline. Experts in argument analysis and evaluation need years of study to evaluate the quality of information. While we do not expect (because this would be unrealistic) that people become experts in argumentation theory, working on these aspects is a way to implement the classical tradition of “critical thinking”. Developing competencies in argumentation theory strengthens individuals as critical thinkers. Indeed, Siegel explains, “a critical thinker is a thinker who can assess claims and make judgments based on reasons, and who understands and conforms to principles governing the evaluation of the force of these arguments” [27]. Basing education and training initiatives on argumentation theory prepares individuals to become more competent in those abilities that are necessary for successful decision-making [28,29,30]. This is a way to encourage individuals to look for evidence and to ask the right questions to scrutinize claims, points of view, or what is presented as evidence.

Thus, our broader aim here is to call for concrete and specific initiatives to empower individuals in information appraisal. This is rather urgent, and there is a whole tradition of theories, models, and tools that can assist in doing this. It is a matter of thinking how to implement the study of critical thinking and argumentation theory in education and training programs, with a focus on the provisions of criteria and standards to assess the quality of information. Of course, expressing one’s opinion is a fundamental right, but the ability to evaluate information should be seen as essential to avoid suboptimal decision-making.

Second, health institutions can play a significant role in reinforcing critical health literacy. For decades, health promotion and disease prevention have been driven by an approach of telling people what is good/bad for their health (for instance, “eating fruits and vegetables is good” and “smoking is bad”) instead of empowering them to deal with the concurring information that stands out from the informational mainstream but might be more appealing, as it offers easier, less strenuous, or more rewarding alternatives. The pitfalls of this one-sided approach have become evident during the pandemic. The global COVID-19 health crisis has shown that this type of top-down approach to health styles sometimes has little impact on people. People are exposed to so much health information from different traditional and alternative sources that, as we argue, empowering critical thinking skills provides the best guidance. By following this approach, the World Health Organization is already actively working to empower governments and institutions to manage infodemics, including informational overload and dis-/misinformation [8]. Building critical health literacy and providing science education plays a significant role in this framework.

Third, this paper points to the benefits of efforts that target the educational system. Disciplines such as epistemology, philosophy of science, critical thinking, and scientific thinking can inform educational programs in different settings and different levels of education. Thus, for instance, school-based programs could entail specific sections that lay the foundations of scientific thinking and health information quality. A primary focus should be on what evidence is, how it differs from opinions, and the difference between causality and correlation. Similarly, patient education programs, instead of focusing on information provision, could be based on argumentation theory, so to provide patients with critical thinking skills that are useful in making informed decisions concerning their health.

At this stage, we also like to acknowledge one important arising limitation. This paper is conceptual. More work on the presentation of best practices of education and training is needed. Additionally, this paper focuses on argumentation theory. It should be complemented by work focusing on the so-called biases and heuristics well-developed in the cognitive sciences [31]. While we claim that the ability to evaluate health information would be an asset for individuals’ decision-making, we also have to acknowledge that this ability can be negatively impacted by the use of heuristics in critical thinking. Thus, for instance, people may evaluate information wrongly because they have a bias. For a discussion on the role of heuristics in critical thinking, we refer to a previous publication [32]. On the link between argumentation skills and heuristics, we plan to conduct further research.

## 5. Conclusions

This paper contributes to the operationalization of what critical health literacy entails by looking at the structure and soundness of arguments. Indeed, since false and unsupported claims in health information might pose a risk to individuals’ health, it is fundamental to equip them with the necessary means to assess the credibility and correctness of the claims that confront them. As a well-developed field of research, argumentation theory proposes several concepts and tools to assist with these challenges. Moreover, some of argumentation theory’s main aspects are the basis of current handbooks and courses in critical thinking that have entire sections dedicated to evaluating arguments. The development of argumentation skills requires knowledge and intensive training, which might be an implementational impediment on a larger scale, e.g., for whole societal groups or communities. Yet, some main concepts could inform educational interventions within public health frameworks and help disseminate a general understanding of good versus bad instances of argumentation. As a significant part of information nowadays has a persuasive nature, guiding the public understanding of argumentation and its principles is a major step toward consolidating critical literacy skills. In an information society, where freedom of speech is a crucial value, providing some normative guidance to individuals is a vital step towards empowering a free and reasonable choice about what to believe or not. Francis Bacon once said: “read not to contradict and confute; nor to believe and take for granted… but to weigh and consider.” [33] An argumentation theory-based education and training can help to achieve this goal.

## Data Availability

Not applicable.

## References

[1] WHO Director-General’s Opening Remarks at the Media Briefing on COVID-19-5 March 2020. https://www.who.int/dg/speeches/detail/who-director-general-s-opening-remarks-at-the-media-briefing-on-covid-19-5-march-2020.

[2] Eysenbach G., Powell J., Kuss O., Sa E.R. (2002). Empirical studies assessing the quality of health information for consumers on the world wide web: A systematic review. JAMA.

[3] Cline R.J., Haynes K.M. (2001). Consumer health information seeking on the Internet: The state of the art. Health Educ. Res..

[4] Allington D., Duffy B., Wessely S., Dhavan N., Rubin J. (2020). Health-protective behaviour, social media usage and conspiracy belief during the COVID-19 public health emergency. Psychol. Med..

[5] Brennen J.S., Simon F., Howard P.N., Nielsen R.K. (2020). Types, sources, and claims of COVID-19 misinformation. Reuters Inst..

[6] Burel G., Farrell T., Mensio M., Khare P., Alani H. Co-spread of Misinformation and Fact-Checking Content during the Covid-19 Pandemic. Proceedings of the 12th International Conference on Social Informatics.

[7] Carmi E., Yates S.J., Lockley E., Pawluczuk A. (2020). Data citizenship: Rethinking data literacy in the age of disinformation, misinformation, and malinformation. Internet Policy Rev..

[8] Call for Action: Managing the Infodemic. https://www.who.int/news/item/11-12-2020-call-for-action-managing-the-infodemic.

[9] Rubinelli S., Schulz P.J., Nakamoto K. (2009). Health literacy beyond knowledge and behaviour: Letting the patient be a patient. Int. J. Public Health.

[10] Diviani N., Obrenovic J., Montoya C.L., Karcz K. (2020). Disentangling health information appraisal competence: Results from an interdisciplinary scoping review and online consultation among Swiss stakeholders. PLoS ONE.

[11] Chinn D. (2011). Critical health literacy: A review and critical analysis. Soc. Sci. Med..

[12] Diviani N. (2019). On the Centrality of Information Appraisal in Health Literacy Research. HLRP Health Lit. Res. Pract..

[13] van Eemeren F.H., Grootendorst R., Kruiger T. (2019). Handbook of Argumentation Theory: A Critical Survey of Classical Backgrounds and Modern Studies.

[14] van Eemeren F.H., Grootendorst R., Jacobs C.S., Jackson S.A. (2002). Reconstructing Argumentative Discourse.

[15] van Eemeren F.H., Grootendorst R., Eemeren F.H. (2004). A Systematic Theory of Argumentation: The Pragma-Dialectical Approach.

[16] Rubinelli S. (2013). Rational versus unreasonable persuasion in doctor-patient communication: A normative account. Patient Educ. Couns..

[17] Rubinelli S., Henkemans A.F.S. (2014). Argumentation and Health.

[18] Walton D. (2005). Fundamentals of Critical Argumentation.

[19] Walton D. (2015). Argument Evaluation and Evidence.

[20] Jiménez-Aleixandre M.P., Puig B. (2012). Argumentation, evidence evaluation and critical thinking. Second International Handbook of Science Education.

[21] Van Eemeren F.H., Grootendorst R., Meuffels B. (1989). The skill of identifying argumentation. J. Am. Forensic Assoc..

[22] Cacioppo J.T., Petty R.E. (1986). The elaboration likelihood model of persuasion. Communication and Persuasion.

[23] Walton D., Reed C., Macagno F. (2008). Argumentation Schemes.

[24] van Eemeren F.H., Garssen B., Labrie N. (2021). Argumentation between Doctors and Patients: Understanding Clinical Argumentative Discourse.

[25] van Eemeren F.H., Grootendorst R. (2016). Argumentation, Communication, and Fallacies: A Pragma-Dialectical Perspective.

[26] van Eemeren F.H., Henkemans A.F.S. (2016). Argumentation: Analysis and Evaluation.

[27] Siegel H. (1980). Critical thinking as an educational ideal. The Educational Forum.

[28] Andrews R. (2015). Critical thinking and/or argumentation in higher education. The Palgrave Handbook of Critical Thinking in Higher Education.

[29] Hitchcock D. (2017). On Reasoning and Argument.

[30] Hyytinen H., Toom A., Shavelson R.J. (2019). Enhancing Scientific Thinking Through the Development of Critical Thinking in Higher Education. Redefining Scientific Thinking for Higher Education.

[31] Tversky A., Kahneman D. (1974). Judgment under uncertainty: Heuristics and biases. Science.

[32] Rubinelli S., Diviani N., Fiordelli M., Bahri P. (2020). The Cognitive and Behavioral Sciences. Communicating about Risks and Safe Use of Medicines: Real Life and Applied Research.

[33] Bacon F. (1888). Essays.

